# 

**Published:** 1855-12

**Authors:** 


Books Received.

Dickson's Elements of Medicine, from Blanchard & Lea, Philadelphia.

Simpson's Obstetrical Papers and Memoirs. Philadelphia: J. B. Lippincott & Co.

Jackson's Letters to a Young Physician. Boston: Phillips, Sampson & Co. N. Y.: J. C. Derbey, 1855.

Carpenter's Human Physiology. Blanchard & Lea: Phila.

Stokes on the Heart and Aorta. Lindsay & Blakiston: Phila.

Lehman's Physiological Chemistry. Blanchard & Lea: Phila.

Johnson on Epidemic Cholera. Sindsay & Blakiston: Phila.

Fransactions of the American Medical Association, Vol. VIII.: 1855. From committee of publications.

The following is the table of Contents and Minutes of the eighth annual meeting of the Association.

Report of the Committee of Publications.

Report of Treasurer.

Address of Charles A. Pope, Professor of the Association.

Report on the Disease of Missouri and Iowa.

The report of the committee on the hygrometrical state of the atmosphere in various localities, and its influence on Health. By Prof. S. B. Hunt·

Deformities after Fractures. By Prof. Frank H. Hamilton.

Report on the Diet of the Sick. By Chas. Hooker.

The pathology, causes, symptoms and treatment of Scrofula. By W. H. Byford.

Report of Committee on the means of preserving milk, and on the influence of Pregnancy and menstruation on the composition and nutritive qualities of that fluid. By Prof. N. S. Davis.

Report of the Committee on Dysintery.

The Effects of Alcoholic Liquors in Health and disease. By Prof. R. D. Mussey.

Sketch of the Caustic Pulverizer. By. R. H. Thomas.

Prize Essay—Statistics of Placenta Prævia. By J. D. Trask.

Plan of Organization of the American Medical Association.

Officers of the Association for 1855.

List of permanent members. J.

---

### EDITORIAL.

*Card of the Committee on Prize Essays of the American Medical Association.*—At a meeting of the American Medical Association held in Philadelphia, May 1855, the undersigned were

*To Subscribers.*—The present number closes volume four of the new series of the *North-Western Medical and Surgical Journal.* In it we enclose bills to a large number of subscribers who stand indebted on our books for one or more years; and we hope those to whom they are sent will give them prompt attention. If by mistake of the Clerk or by failure in the transmission of money by mail, any receive bills who have previously sent the money to pay the same, they will much oblige us by giving immediate notice of the fact, and we will most cheerfully correct all errors. We are willing to incur all the risk and losses by mail where the letters are plainly directed to the address of the undersigned.

We wish to give a fair notice also, that every name on our subscription list, which stands indebted to us for more than *one year*, on the first day of February 1856 will be stricken off. We have placed the printing of the new volume, commencing with the January number, in the hands of a Printer of tried experience and fidelity; and who is pledged to use better type, better paper, and greater punctuality, than has been used in times past. Our object is to make our Journal as valuable, in every respect, to the physicians of the North-West, as any other on this continent.

To secure this, we shall bestow upon it our best judgement and energies; and we cordially invite the co-operation and *contributions* of our readers.

N. S. Davis.

